# Refinement and Neutralization Evaluation of the F(ab’)_2_ Type of Antivenom against the Deadly Jellyfish *Nemopilema nomurai* Toxins

**DOI:** 10.3390/ijms222312672

**Published:** 2021-11-24

**Authors:** Rongfeng Li, Huahua Yu, Aoyu Li, Chunlin Yu, Pengcheng Li

**Affiliations:** 1 Key Laboratory of Experimental Marine Biology, Center for Ocean Mega-Science, Institute of Oceanology, Chinese Academy of Sciences, Qingdao 266071, China; yuhuahua@qdio.ac.cn (H.Y.); aoyulee1227@163.com (A.L.); Yuchunlin17@mails.ucas.ac.cn (C.Y.); 2 Laboratory for Marine Drugs and Bioproducts, Pilot National Laboratory for Marine Science and Technology (Qingdao), No. 1 Wenhai Road, Qingdao 266237, China; 3 College of Earth and Planetary Sciences, University of Chinese Academy of Sciences, Beijing 100049, China

**Keywords:** jellyfish, *Nemopilema nomurai*, antivenom, NnTXs, IgG-AntiNnTXs, F(ab’)_2_-AntiNnTXs

## Abstract

Jellyfish stings threaten people’s health and even life in coastal areas worldwide. *Nemopilema nomurai* is one of the most dangerous jellyfish in the East Asian Marginal Seas, which not only stings hundreds of thousands of people every year but also is assumed to be responsible for most deaths by jellyfish stings in China. However, there is no effective first-aid drug, such as antivenoms, for the treatment of severe stings by *N. nomurai* to date. In this study, we prepared a *N. nomurai* antiserum from rabbits using inactivated *N. nomurai* toxins (NnTXs) and isolated the IgG type of antivenom (IgG-AntiNnTXs) from the antiserum. Subsequently, IgG-AntiNnTXs were refined with multiple optimizations to remove Fc fragments. Finally, the F(ab’)_2_ type of antivenom (F(ab’)_2_-AntiNnTXs) was purified using Superdex 200 and protein A columns. The neutralization efficacy of both types of antivenom was analyzed in vitro and in vivo, and the results showed that both IgG and F(ab’)_2_ types of antivenom have some neutralization effect on the metalloproteinase activity of NnTXs in vitro and could also decrease the mortality of mice in the first 4 h after injection. This study provides some useful information for the development of an effective antivenom for *N. nomurai* stings in the future.

## 1. Introduction

**Figure 1:**
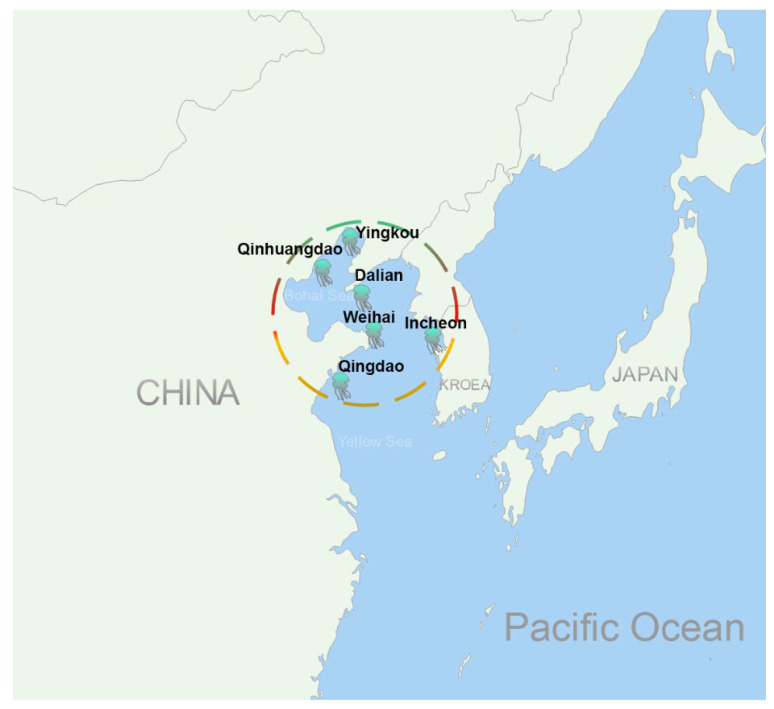
Distribution of death reports by jellyfish *N. nomurai* stings in recent years. The locations with the jellyfish symbols represent where deaths due to jellyfish stings were reported.

Jellyfish stings are a serious threat to people’s health and even life at the seaside worldwide. Every year, a huge number of people suffer pain, itching, redness, swelling, and inflammation from jellyfish stings. Some victims even die from the stings of some venomous species of jellyfish, such as the box jellyfish *Chironex fleckeri*, *Chiropsalmus quadrigatus*, *Irukandji*, and the Portuguese man-of-war *Physalia physalis*. It was reported that about 20 to 50 deaths occurred annually in Thailand and the Philippines seaside and more than 63 victims died in Australian waters up to 1994 caused by box jellyfish stings [1,2,3,4]. *Nemopilema nomurai*, a big and venomous jellyfish with a body size of 2 m maximum bell diameter and 200 kg wet weight, often blooms in the East Asian Marginal Seas (i.e., the Bohai, Yellow, East China, and Japan Seas) [5,6]. This jellyfish has hundreds of thousands of victims every year and is assumed to be responsible for most of the deaths by jellyfish stings in these areas [7]. Since 2006, at least 11 deaths have been reported in China and Korea (Figure 1), including 63.6% females, 36.4% males, and 36.4% children.

Most of the deaths were due to acute pulmonary edema after jellyfish *N. nomurai* stings (Table 1). In addition, jellyfish *N. nomurai* toxins could also cause mice kidney and liver damage, including renal glomerular swelling, renal vesicle stricture, renal tubules dilatation, and hepatic blood sinusoid dilatation [8]. The toxicity of jellyfish *N. nomurai* toxins is strong and multitargeted in the body. However, no effective antidote is available for this jellyfish sting to date.

Jellyfish venom is mainly composed of various toxins, including phospholipase A_2_, hemolysin, metalloprotease, potassium channel inhibitor, and C-type lectin [11,12,13,14,15,16,17,18]. Most of the jellyfish toxins are proteins with a large molecular weight, which may have good antigenicity to produce antibodies. These antibodies, as antivenoms, may be developed as first-aid medicine to deal with severe jellyfish stings. Antivenoms, especially snake antivenoms, have already been successfully used for the treatment of poisonous animals’ bites or stings for many years. Although antivenoms are effective in neutralizing the toxicity of animal toxins, side effects may also occur, including serum sickness, shortness of breath, and anapylaxis [19]. An antiserum comprises antibodies (immunoglobulin G (IgG)), plasma proteins, and peptides as well, which may also cause an immune response when injected into the body. IgG is composed of two fragments of antigen-binding domains (Fab) and a fragment of crystallizable domain (Fc), but only the Fab domain interacts with the antigen [20]. In addition, most antivenoms for medical use are produced by animals, such as horses, and the heterogenous IgG or Fc domain may also have immunogenicity in the body and cause some serum sicknesses [21]. So, purification of IgG from the antiserum and further removal of the Fc domain from IgG may be better choices to decrease the potential side effects of antivenoms [22,23].

In this study, we prepared an antivenom for jellyfish *N. nomurai* stings in rabbits using inactivated *N. nomurai* toxins as the antigen. IgG-AntiNnTXs were isolated from the rabbits’ serum and then refined to the F(ab’)_2_ type of antivenom (F(ab’)_2_-AntiNnTXs) by pepsin to remove the Fc domain of the IgG-AntiNnTXs. The neutralization efficacy of these two types of antivenom was compared and analyzed both in vitro and in vivo. Both types of antivenom showed some neutralization effect on the toxicities of NnTXs. This study provides some useful information for the development of effective jellyfish antivenoms in the future.

## 2. Results

### 2.1. Preparation and Isolation of IgG-AntiNnTXs

**Figure 2:**
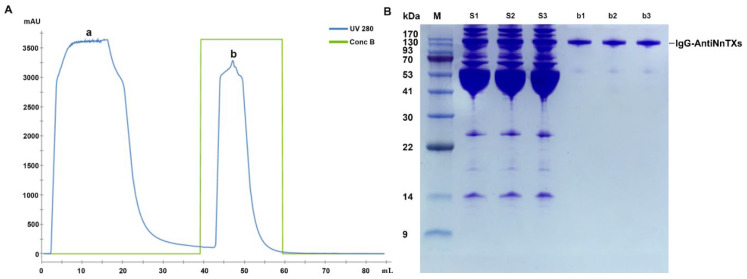
Isolation of IgG-AntiNnTXs from the antiserum. (**A**) Affinity chromatogram of IgG-AntiNnTXs with a protein A column. Peak a: flow through; peak b: IgG-AntiNnTXs containing elution by buffer B (pH = 3.0, 100 mM glycine). (**B**) SDS-PAGE profile of the fractions from the protein A purification. M: protein markers; S1, S2, and S3: antiserum; b1, b2, and b3: fraction of peak b.

The crude antiserum contained IgG-AntiNnTXs and many other serum proteins. Protein A resin could specifically bind the Fc domain of IgG, so it could isolate the IgG-AntiNnTXs from the antiserum with high affinity, and the other proteins were removed in flow-through (Figure 2A). SDS-PAGE showed that the purity of the IgG-AntiNnTXs was good, with an obvious protein band between 130 kDa and 170 kDa (Figure 2B). This antibody was good enough for further refinement and neutralization assay of the toxicity of NnTXs.

### 2.2. Refinement of F(ab’)_2_ Fragments of IgG-AntiNnTXs

**Figure 3:**
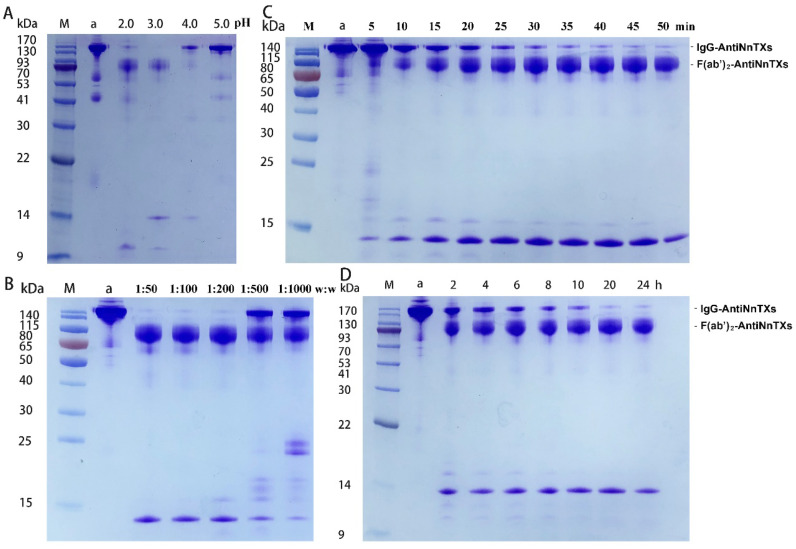
SDS-PAGE analysis of the optimal condition screen for the pepsin digestion of IgG-AntiNnTXs. (**A**) The effect of pH on the pepsin digestion of IgG-AntiNnTXs at 37 °C. M: protein marker; a: IgG-AntiNnTXs; 2.0: pH = 2.0; 3.0: pH = 3.0; 4.0: pH = 4.0; 5.0: pH = 5.0. (**B**) The effect of mass ratio (W_pepsin_:W_IgG-AntiNnTXs_) on the pepsin digestion of IgG-AntiNnTXs at 37 °C. M: protein marker; a: IgG-AntiNnTXs; 1:50: 1:100, 1:200, 1:500, 1:1000: mass ratios of pepsin to IgG-AntiNnTXs. (**C**) The effect of reaction time on the pepsin digestion of IgG-AntiNnTXs at 37 °C. M: protein marker; a: IgG-AntiNnTXs; 5, 10, 15, 20, 25, 30, 35, 40, 45, and 50 min: digestion time. (**D**) The effect of reaction time on the pepsin digestion of IgG-AntiNnTXs at 4 °C. M: protein marker; a: IgG-AntiNnTXs; 2, 4, 6, 8, 10, 20, 24 h: digestion time.

The optimization of digestion conditions is crucial to prepare F(ab’)_2_-AntiNnTXs. Figure 3 displays the SDS-PAGE profiles of IgG-AntiNnTXs digests under different conditions. Figure 3A shows that the AntiNnTXs could be digested by pepsin much faster at pH 2.0 or pH 3.0 than at pH 4.0 or 5.0. Almost all the IgG-AntiNnTXs could be digested at pH 2.0 or pH 3.0 at 37 °C for 60 min. Figure 3B shows that a ratio of W_pepsin_:W_IgG-AntiNnTXs_ = 1:50, 1:100, or 1:200 could digest all the IgG-AntiNnTXs at pH 3.0 at 37 °C in 60 min. However, a ratio of W_pepsin_:W_IgG-AntiNnTXs_ = 1:500 or 1:1000 was not effective enough for the digestion of IgG-AntiNnTXs. Figure 3C shows that most of the IgG-AntiNnTXs could be digested in 40 min to 50 min at pH 3.0 at 37 °C. Figure 3D shows that IgG-AntiNnTXs also could be digested at a ratio of W_pepsin_:W_IgG-AntiNnTXs_ = 1:20 at 4 °C in 20 to 24 h.

### 2.3. Purification of F(ab’)_2_-AntiNnTXs by Size-Exclusion Chromatography

**Figure 4:**
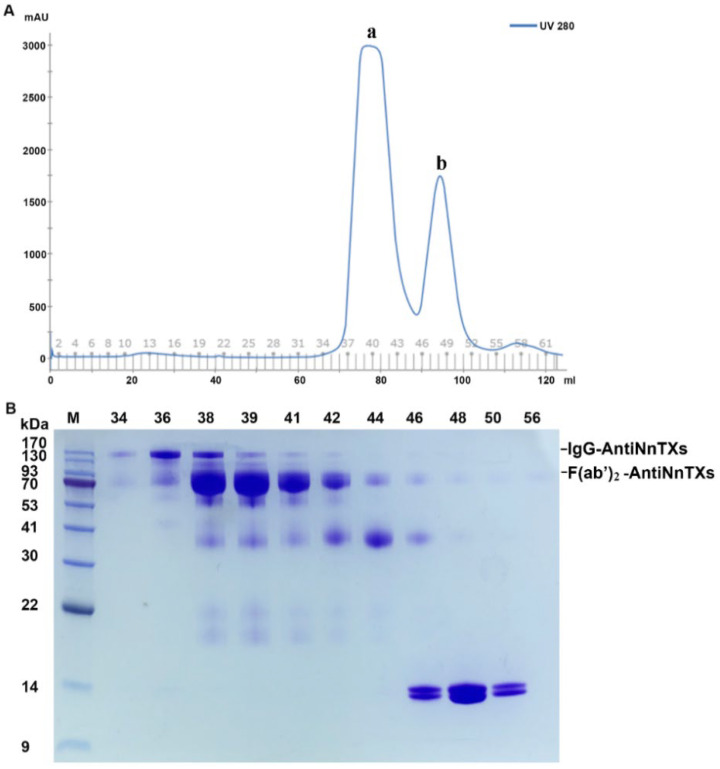
Purification of F(ab’)_2_-AntiNnTXs from the pepsin digests of IgG-AntiNnTXs. (**A**) Superdex 200 purification of F(ab’)_2_-AntiNnTXs from the pepsin digests of IgG-AntiNnTXs. (**B**) SDS-PAGE profile of the fractions from the size-exclusion chromatography purification. M: marker; 34, 36, 38, 39, 41, 42, 44, 46, 48, 50, 56: tube number of fractions.

IgG-AntiNnTXs digests contained undigested IgG-AntiNnTXs, F(ab’)_2_-AntiNnTXs, Fc fragments, and pepsin. The digests were purified by size-exclusion chromatography using HiLoad Superdex 200 16/60 according to the difference in their molecular weights. Figure 4A,B shows that the Fc fragments and pepsin could be removed from the digests using this column.

### 2.4. Purification of F(ab’)_2_-AntiNnTXs by Protein A

**Figure 5:**
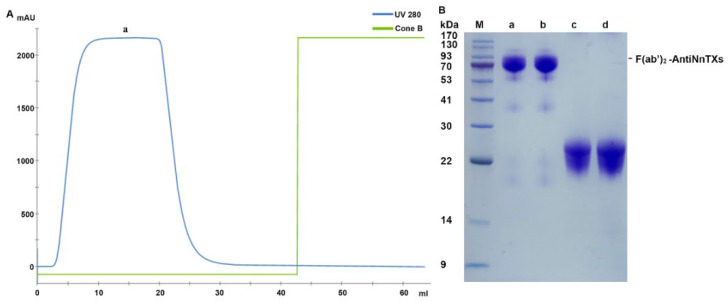
Affinity purification of F(ab’)_2_-AntiNnTXs. (**A**) Protein A purification chromatogram of F(ab’)_2_-AntiNnTXs. (**B**) SDS-PAGE profile of the fractions from the protein A purification. M: marker; a, b, and F(ab’)_2_-AntiNnTXs under the non-reduced condition; c, d, and F(ab’)_2_-AntiNnTXs under the reduced condition.

The undigested IgG-AntiNnTXs and F(ab’)_2_-AntiNnTXs were still in the mixture of peak a. Therefore, a second purification was performed using a protein A column to remove the undigested IgG-AntiNnTXs from the mixture. Figure 5 shows that a high purity of F(ab’)_2_-AntiNnTXs was achieved after this affinity chromatography with a molecular weight of ~90 kDa.

### 2.5. In Vitro Neutralization Assay of Metalloproteinase Activity

**Figure 6:**
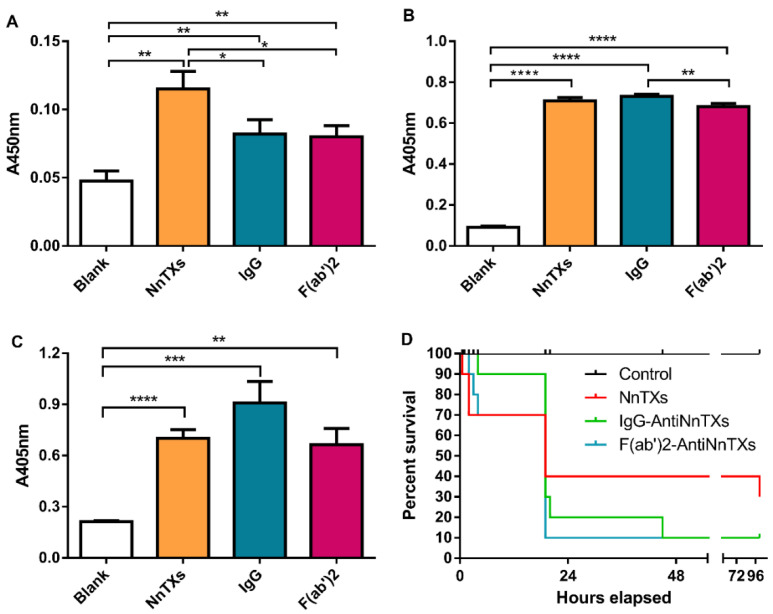
Neutralization assay of antivenoms against the toxicities of NnTXs. (**A**) Neutralization assay of antivenoms against the metalloprotease activity of NnTXs in vitro, (**B**) neutralization assay of antivenoms against the hemolytic activity of NnTXs in vitro, and (**C**) Neutralization assay of antivenoms against the PLA_2_ activity of NnTXs in vitro. * *p* < 0.05; ** *p* < 0.03; *** *p* < 0.0003; **** *p* < 0.0001; *n* = 3. (**D**) Neutralization assay of IgG-AntiNnTXs and F(ab’)_2_-AntiNnTXs against the lethality of NnTXs in vivo (*n* = 10). Control: injection of dialysis buffer; NnTXs: injection of NnTXs; IgG: injection of IgG-AntiNnTXs neutralized NnTXs; F(ab’)_2_: injection of F(ab’)_2_-AntiNnTXs neutralized NnTXs.

Metalloproteinases are the main components of NnTXs [11]. The inhibitory effect on the metalloproteinase activity of NnTXs is a good indicator of the neutralization efficacy of the NnTXs’ antivenom in vitro. NnTXs showed strong metalloproteinase activity in Figure 6A, and both IgG-AntiNnTXs and F(ab’)_2_-AntiNnTXs exhibited a significant neutralization effect on the metalloproteinase activity of NnTXs. However, no significant difference was observed between IgG-AntiNnTXs and F(ab’)_2_-AntiNnTXs.

### 2.6. In Vitro Neutralization Assay of Hemolytic Activity

Hemolysin is widely distributed in different jellyfish venoms. The inhibitory effect on the hemolytic activity of NnTXs is an important factor for evaluating the neutralization efficacy of the NnTXs’ antivenom in vitro. Figure 6B shows that NnTXs had strong hemolytic activity in erythrocytes. However, neither IgG-AntiNnTXs nor F(ab’)_2_-AntiNnTXs had any inhibitory effect on the hemolytic activity of NnTXs.

### 2.7. In Vitro Neutralization Assay of PLA_2_ Activity

Phospholipase is one of the key lethal toxins in the venom of jellyfish *N. nomurai* and may play an important role in the lethality of NnTXs. Figure 6C shows that NnTXs had obvious PLA_2_ activity. However, IgG-AntiNnTXs and F(ab’)_2_-AntiNnTXs neutralized NnTXs still exhibited strong PLA_2_ activity, which means that neither IgG-AntiNnTXs nor F(ab’)_2_-AntiNnTXs have any inhibitory effect on the PLA_2_ activity of NnTXs.

### 2.8. In Vivo Neutralization Assay

The in vivo neutralization of these two types of antivenom was assayed using mice (Figure 6D). Both IgG-AntiNnTXs and F(ab’)_2_-AntiNnTXs could obviously decrease the mortality of mice in the first 4 h after injection. However, the 4-day survival rate of both IgG-AntiNnTXs and F(ab’)_2_-AntiNnTXs was only 20%, even lower than that of NnTXs (30%), which suggested that these two types of antivenom showed some neutralization effect on NnTXs but also have obvious side effects or toxicity.

### 2.9. LC-MS/MS and Bioinformatics Analysis

**Figure 7:**
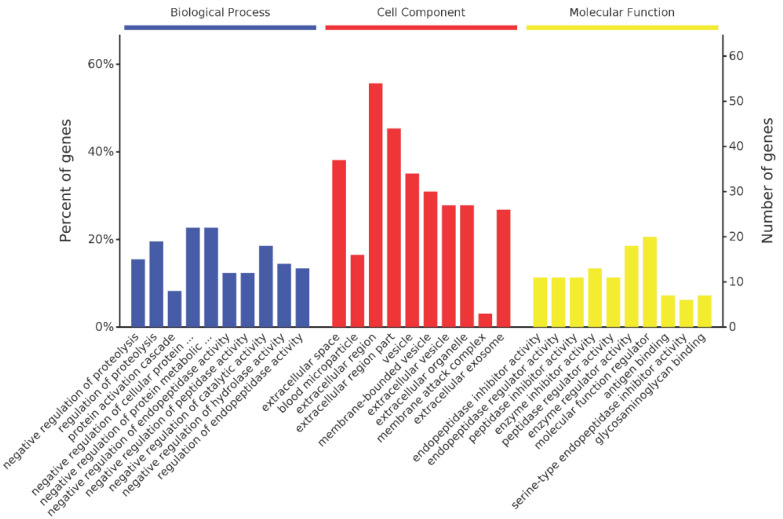
LC-MS/MS and GO analysis of NnTXs antiserum. All the identified proteins were classified as cellular component, biological process, or molecular function.

A total of 116 homologous proteins were matched in the database *Oryctolagus cuniculus* using LC-MS/MS analysis (Figure 7), including the Ig kappa-b4 chain C region, Ig kappa chain V region AH80-5, and metalloproteinase inhibitor 3. Gene Ontology (GO) analysis of all the identified proteins was performed to better understand the antivenom. All the identified proteins were classified as molecular function, biological process, or cellular component. Molecular function analysis showed that most of the proteins function as protein inhibitors, enzyme regulators, or glycosaminoglycan receptors. The antivenom is composed of plenty of antibodies and functions as an inhibitor of toxins. Biological process analysis showed that most of these proteins participate in negative regulation of proteolysis, catalytic activity, and hydrolase activity.

## 3. Discussion

Jellyfish stings have already become a serious threat to human health or even life globally. Every year, a huge number of people get stung by jellyfish; unfortunately, some of whom die in a short time due to a lack of effective rescue. Although some treatments, such as vinegar, alumen solution, or seawater rinsing, are recommended as first aid to allay the symptoms of jellyfish stings [22,24,25], they are not effective enough to save the lives of those who get a severe jellyfish sting. Therefore, a much more effective medication will be needed for an emergency. An antivenom is thought to be an effective medicine for the treatment of venomous animal bites or stings for many years, especially for snake bites, spider stings, scorpion stings, etc.

*Nemopilema nomurai* is a deadly jellyfish and has killed many people in China and Korea. Based on the reports, all the death cases were distributed in the Bohai Sea and the Yellow Sea, where jellyfish *N. nomurai* often bloom in the summer. Although only 11 death cases were reported in this study, the real number will be much more than that. Many deaths were not reported or only reported to be caused by jellyfish stings but without any information about which species of jellyfish. Moreover, *N. nomurai* is the most venomous jellyfish and is assumed to be responsible for most of the deaths due to jellyfish stings in the North China Sea [7]. The toxicity of *N. nomurai* venom is strong and can cause acute pulmonary edema, multiple-organ failure, or even death in a few hours [8,26]. However, there is no specific antivenom for this jellyfish venom to date in such areas. It is important to develop a jellyfish *N. nomurai* sting antivenom for emergent use.

An antivenom is composed of many antibodies for various toxins, which can neutralize the toxins by binding them with the Fab region of IgG. Nowadays, there are three types of antivenom, including IgG type, F(ab’)_2_ type, and Fab type [22,27,28]. The IgG type and F(ab’)_2_ type of antivenoms are the dominant commercial types. The only commercial jellyfish antivenom, Commonwealth Serum Laboratories™ (CSL) box jellyfish antivenom, is also an IgG type. Our previous study showed that the F(ab’)_2_ type of jellyfish *Cyanea nozakii* antivenom is more effective than the IgG type of antivenom and much more effective than the Fab type of antivenom [22]. Therefore, in this study, the IgG type of jellyfish *N. nomurai* antivenom (IgG-AntiNnTXs) was prepared and then refined to the F(ab’)_2_ type of antivenom (F(ab’)_2_-AntiNnTXs) by pepsin under optimized conditions. The neutralization assay results showed that both IgG-AntiNnTXs and F(ab’)_2_-AntiNnTXs had some neutralization effect on the metalloproteinase activity of NnTXs in vitro. LC-MS/MS and GO analysis of identified proteins from the antiserum of NnTXs showed that this antiserum contains many proteinase inhibitors, such as metalloproteinase inhibitors. That may be the reason why the antivenom has a neutralization effect on the metalloproteinase activity of NnTXs. However, neither IgG-AntiNnTXs nor F(ab’)_2_-AntiNnTXs showed any inhibitory effect on the in vitro hemolytic and PLA_2_ activity of NnTXs. Phospholipase, potassium channel inhibitor, and hemolysin might be the key lethal toxins in NnTXs [29]. This may be why both types of antivenom could not increase the 96 h survival rate of mice and even caused higher mortality. It was reported that the crude antiserum using native *N. nomurai* venom as an antigen shows a good neutralization effect on in vitro toxicity. In addition, this antiserum could increase the survival rate of mice to 25% in 24 h at a venom: antiserum ratio (*w*/*v*) of 1:5 but without any protective effect at a ratio lower than 1:2.5 [30]. The antigen used for immunization is important. The current extracted jellyfish venom using sonication or glass bead disruption [31,32] contains various toxins and many other nontoxic proteins. These nontoxic proteins may also produce antibodies during immunization, which may affect the neutralization efficiency and even be harmful to mice. In addition, using inactivated toxins as antigens are much safer than native toxins for animals to produce antibodies; however, they may be degraded into small peptides in the process of attenuation and lose some immunogenicity. Therefore, a native and much purer jellyfish venom will be tried as an antigen to make a more effective and safer jellyfish *N. nomurai* antivenom. This study is the first to develop the IgG type and the F(ab’)_2_ type of *N. nomurai* antivenom and compare their neutralization efficacy both in vitro and in vivo, which is novel and worth trying and will be helpful to develop an effective first-aid drug for the treatment of jellyfish (*N. nomurai*) stings in the future.

## 4. Materials and Methods

### 4.1. Preparation and Inactivation of Jellyfish N. nomurai Toxins (NnTXs)

Jellyfish *N. nomurai* were captured from the coast of Qingdao, China. Fresh tentacles were excised manually from the jellyfish immediately and then stored at −80 °C. Jellyfish *N. nomurai* toxins (NnTXs) were extracted according to a previous method [8]. Briefly, frozen jellyfish tentacles were autolyzed overnight at 4 °C and then filtered using a plankton net to remove undissolved tentacles. The filtrate was centrifuged at 10,000× *g* for 15 min at 4 °C. The nematocysts were collected and sonicated in prechilled 20 mM Na_2_HPO_4_ at pH 7.0. After centrifugation at 10,000× *g* for 15 min at 4 °C, 100 mL of the supernatant (NnTXs) was mixed with 2 mL of 40% formaldehyde and incubated at 37 °C for a week. Then, 0.5 mL of 40% formaldehyde was added to the mixture and incubated at 37 °C for another week. After that, the formaldehyde was removed by dialysis against 20 mM Na_2_HPO_4_ at pH 7.0. Finally, the sample was centrifugated at 10,000× *g* for 15 min, and the supernatant was filtered with a 0.22 µm filter and stored at −80 °C.

### 4.2. Animal Immunization and Antiserum Preparation

The inactivated *N. nomurai* toxins were used as the antigen to immunize three New Zealand white rabbits (4 M, 2.1 kg). Briefly, 0.7 mg of the antigen with complete Freund’s adjuvant (CFA) was injected into the rabbits for the first immunization. Three weeks later, 0.35 mg of the antigen with incomplete Freund’s adjuvant (IFA) was injected into the rabbits for secondary immunization. After 2 weeks, 0.35 mg of the antigen with IFA was injected into the rabbits for the third immunization, and a small amount of blood was extracted for serum titer measurement using enzyme-linked immunosorbent assay (ELISA). One week later, the last immunization was performed with 0.35 mg of the antigen with IFA. The next day, all the blood was collected, the final antiserum titer was measured using ELISA, and the antiserum was analyzed by SDS-PAGE. All animals received humane care, and the study was approved by the ethics committee of the Institute of Oceanology, Chinese Academy of Sciences.

The ELISA measurement of the antiserum titer was as follows: 100 µL of inactivated NnTXs was diluted in 50 mm pH9.6 Na_2_CO_3_ and coated in a microtiter plate at 4 °C overnight. The coating solution was removed, and the plate was washed three times with 0.05% Tween-20 and 20 mM NaH_2_PO_4_ at pH 7.4 (PBST). The coated NnTXs were then blocked with 5% skim milk at 37 °C for 1 h and then washed three times with PBST. Next, 100 µL of antiserum dilutions was added and incubated at 37 °C for 1 h, followed by washing three times with PBST. Diluted HRP-labeled goat anti-rabbit IgG (H+L) was added and incubated at 37 °C for 45 min. The plates were then washed three times with PBST, and 100 µL of 3′3′5′5′-tetramethyl benidine dihydrochloride (TMB) substrate was added. To stop the reaction, 100 µL of 2 M H_2_SO_4_ was added after 15 min, and absorbance was measured at 450 nm.

### 4.3. Isolation of NnTXs Antibody from Antiserum

NnTXs antibody (IgG-AntiNnTXs) was isolated with a protein A affinity column (GenScript, Piscataway, NJ, USA) from the antiserum using a fast protein liquid chromatogram system ÄKTA pure (GE Healthcare, Chicago, IL, USA). Briefly, the antiserum was mixed with isometric binding buffer A (0.15 M NaCl, pH 7.0, 20 mM Na_2_HPO_4_) and was loaded onto the protein A column. The column was washed with buffer A, and AntiNnTXs were eluted with buffer B (pH = 3.0, 100 mM glycine). The purity of the AntiNnTXs was analyzed using SDS-PAGE.

### 4.4. Refinement of F(ab’)_2_ Fragments of IgG-AntiNnTXs

The optimal condition screen of pepsin digestion of IgG-AntiNnTXs was performed according to a previous method [22]. The optimum pH for the pepsin digestion was determined as follows: IgG-AntiNnTXs were dialyzed at 4 °C overnight against dialysis buffers at pH 2.0, pH 3.0, pH 4.0, and pH 5.0 and 100 mM glycine. IgG-AntiNnTXs were digested at different pH values with equal pepsin at 37 °C for 60 min with three replicates, and the digested IgG-AntiNnTXs were analyzed using SDS-PAGE. The optimum W_pepsin_:W_IgG-AntiNnTXs_ ratio for the pepsin digestion was determined as follows: Pepsin was added to AntiNnTXs to a final ratio of 1:50, 1:100, 1:200, 1:500, or 1:1000. The reaction was carried out at pH 3.0 at 37 °C for 60 min with three replicates, and the digested IgG-AntiNnTXs were analyzed using SDS-PAGE. The optimum time for pepsin digestion was determined as follows: IgG-AntiNnTXs were digested with equal pepsin at pH 3.0 and 37 °C for 5, 10, 15, 20, 25, 30, 35, 40, and 50 min with three replicates. The digested IgG-AntiNnTXs were immediately quenched at 95 °C for 5 min once the reaction finished and were then analyzed using SDS-PAGE. The reaction temperature was evaluated as follows: IgG-AntiNnTXs were digested with equal pepsin with a ratio of W_pepsin_:W_IgG-AntiNnTXs_ = 1:20 at 4 °C for 2, 4, 6, 8, 10, 20, and 24 h. The digested IgG-AntiNnTXs were immediately quenched at 95 °C for 5 min once the reaction finished and were then analyzed using SDS-PAGE.

### 4.5. Purification of F(ab’)_2_-AntiNnTXs

The digestion of IgG-AntiNnTXs was scaled up at pH 3.0 with a mass ratio of W_pepsin_:W_AntiNnTXs_ = 1:20 for 20 h at 4 °C. The digests were concentrated with concentrators (MWCO 10 kDa Millipore, Carrigtwohill, Cork, Ireland) at 7000 ×g at 4 °C and then separated using a HiLoad Superdex 200 16/60 column (GE Healthcare, Chicago, IL, USA) with ÄKTA pure (GE Healthcare, Chicago, IL, USA) using buffer A to remove the Fc domains and pepsins. The first peak containing undigested IgG-AntiNnTXs and F(ab’)_2_-AntiNnTXs was pooled and then further purified with a protein A affinity column (GenScript, Piscataway, NJ, USA) to remove the IgG-AntiNnTXs. During the purification, the purity of IgG-AntiNnTXs was analyzed using SDS-PAGE.

### 4.6. Neutralization Assay of the Antivenoms

#### 4.6.1. In Vitro Neutralization Assay of Metalloproteinase Activity

The neutralization effect of the antivenom on metalloproteinase activity of NnTXs was tested as previously described [33]. In brief, 38 μg NnTXs, 38 μg IgG-AntiNnTXs, or F(ab’)_2_-AntiNnTXs were mixed and incubated at an ice box for 1 h, followed by centrifugation at 10,000 rpm for 15 min at 4 °C. Then, 25 μL supernatant was added to 90 μL of 5 mg/mL of Azocasein in 50 mM Tris-HCl (pH 8.8) and 100 mM NaCl and then incubated at 37 °C for 90 min. The reactions were stopped by adding 200 μL of 0.5 M trichloroacetic acid and then placed at room temperature for 30 min. After centrifugation at 10,000 rpm for 10 min, 150 μL of the supernatant was neutralized with 150 μL of 0.5 M NaOH, and absorbance was then measured at 450 nm. This experiment was conducted with three replicates.

#### 4.6.2. In Vitro Neutralization Assay of Hemolysis Activity

The neutralization effect of the antivenom on the hemolytic activity of NnTXs was assayed using the method previously described [34,35]. Briefly, 38 μg of NnTXs and 38 μg of IgG-AntiNnTXs or F(ab’)_2_-AntiNnTXs were mixed and incubated in an icebox for 1 h, followed by centrifugation at 10,000 rpm for 15 min at 4 °C. Next, 25 μL of the supernatant was added to 200 μL of chicken erythrocyte suspension and incubated at 37 °C for 30 min with isotonic buffer (0.145 M NaCl) as a blank control. After centrifugation at 3000 rpm for 10 min, the hemoglobin released in the supernatant was measured at 405 nm. This experiment was conducted with three replicates.

#### 4.6.3. In Vitro Neutralization Assay of Phospholipase A_2_ (PLA_2_) Activity

The neutralization effect of the antivenom on the PLA_2_ activity of NnTXs was measured with the monodisperse synthetic chromogenic substrate 4-nitro-3-octanoyloxybenzoic acid (NOBA) according to a previous method [26]. In brief, 38 μg of NnTXs and 38 μg of IgG-AntiNnTXs or F(ab’)_2_- AntiNnTXs were mixed and incubated in an icebox for 1 h, followed by centrifugation at 10,000 rpm for 15 min at 4 °C. Then, 25 μL of the supernatant was added to 200 μL of 50 mM Tris-HCl (pH 8.0), 10 mM CaCl_2_, and 100 mM NaCl in a 96-well plate, followed by adding 25 μL of 1 mg/mL of NOBA and incubating at 37 °C for 1 h. Finally, absorbance was measured at 405 nm. This experiment was conducted with three replicates.

#### 4.6.4. In Vivo Neutralization Assay of the Antivenom

In vivo neutralization assay of the lethality of NnTXs was performed according to a method described before [22]. Briefly, SPF-grade KM mice (16–18 g) were used for in vivo neutralization assay. Each group contained 10 mice with 5 males and 5 females. IgG-AntiNnTXs and F(ab’)_2_-AntiNnTXs were dialyzed in 20 mM Tris-HCl (pH 7.0) and 0.15 M NaCl overnight at 4 °C, respectively. IgG-AntiNnTXs or equal mole F(ab’)_2_-AntiNnTXs were incubated with NnTXs at 4 °C for 1 h to neutralize the toxicity of NnTXs. Then, 1 mL of neutralized NnTXs was injected into each mouse using dialysis buffer and NnTXs as the control. The mortality of mice was recorded over the next 4 days. All animal experiments in this study were approved by the ethics committee of the Institute of Oceanology, Chinese Academy of Sciences.

### 4.7. LC-MS/MS and Bioinformatics Analysis of Antivenom

To analyze the components of the antivenom, LC-MS/MS was used according to a previous method [22]. The antiserum was run in SDS-PAGE, and the whole lane containing the antiserum was cut off and washed twice with water. The gel was destained with 25 mM NH_4_HCO_3_ and 50% acetonitrile at room temperature for 30 min, followed by dehydration with 50% acetonitrile for 30 min and 100% acetonitrile for 30 min. Then, it was treated with 10 mM DTT and 25 mM NH_4_HCO_3_ at 57 °C for 1 h, followed by 50 mM iodoacetamide, 25 mM NH_4_HCO_3_, 25 mM NH_4_HCO_3_, 10 mM DTT, 25 mM NH_4_HCO_3_, 50 mM iodoacetamide, and 25 mM NH_4_HCO_3._ The gel was then rehydrated in 10 µL of 0.02 µg/µL of trypsin in 25 mM NH_4_HCO_3_, and 10% acetonitrile and 20 µL of cover solution were added for digestion for 16 h at 37 °C. The gel was extracted with 5% TFA and 67% acetonitrile at 37 °C for 30 min. The extracted peptides and the supernatant were combined and completely dried. The dried peptides were resuspended with 0.1% formic acid and 2% acetonitrile and then loaded onto a C_18_ nanoLC trap column (100 µm × 3 cm, C_18_, 3 µm, 150 Å). The column was washed with 0.1% formic acid and 2% acetonitrile for 10 min and then analyzed with a ChromXP C_18_ column (75 μm × 15 cm, C_18_, 3 μm 120 Å) by an elution gradient of 5–35% acetonitrile and 0.1% formic acid for 90 min. Data were acquired using a Triple TOF 5600 System (AB SCIEX, Framingham, MA, USA) with a Nanospray III source and a pulled quartz tip as the emitter (New Objectives, Woburn, MA, USA). Survey scans were acquired in 250 ms for information-dependent acquisition. All proteins were identified based on combined MS and MS/MS spectra with ≥95% confidence interval scores in the MASCOT V2.3 search engine (Matrix Science, Boston, MA, USA) in the *Oryctolagus cuniculus* database. All identified proteins were annotated in the non-redundant protein GO database.

### 4.8. Statistical Analysis

All the results were expressed as the mean ± SD. Statistically significant differences between groups were considered only when *p* < 0.05.

## 5. Conclusions

In this study, we prepared the IgG type of jellyfish *N. nomurai* antivenom, IgG-AntiNnTXs, and then refined it to the F(ab’)_2_ type of antivenom, F(ab’)_2_-AntiNnTXs, using pepsin under optimized conditions. The neutralization efficacy of these two types of antivenom was compared and analyzed in vitro and in vivo. The results showed that both IgG and F(ab’)_2_ types of antibodies have some neutralization effect on the metalloproteinase activity of NnTXs in vitro. Both types of antivenom could decrease the mortality of mice in the first 4 h after injection. The neutralization efficacy of the IgG type of jellyfish *N. nomurai* antivenom was better than that of the F(ab’)_2_ type of antivenom. This study provides some useful information for the development of a more effective antivenom for jellyfish *N. nomurai* stings in the future.

## Figures and Tables

**Table 1:** Death reports by jellyfish *N. nomurai* stings in recent years.

No.	Year	Gender	Age	Location	Death Reasons
1	2015	Female	4	Dalian, China	Unknown
2	2014	Male	20	Qingdao, China	Expiratory dyspnea
3	2014	Male	31	Dalian, China	Acute pulmonary edema
4	2013	Male	8	Qinhuangdao, China	Acute pulmonary edema
5	2012	Female	47	Qinhuangdao, China	Acute pulmonary edema, allergy
6	2012	Female	8	Incheon, Korea	Acute pulmonary edema [9]
7	2007	Male	50	Weihai, China	Acute pulmonary edema [10]
8	2007	Female	47	Weihai, China	Acute pulmonary edema [10]
9	2007	Female	22	Weihai, China	Acute pulmonary edema [10]
10	2006	Female	9	Yingkou, China	Unknown
11	2006	Female	35	Yingkou, China	Multiple-organ failure
